# Erectile function preservation after salvage radiation therapy for biochemically recurrent prostate cancer after prostatectomy: Five-year results of the SAKK 09/10 randomized phase 3 trial

**DOI:** 10.1016/j.ctro.2024.100786

**Published:** 2024-04-25

**Authors:** Daniel R. Zwahlen, Christina Schröder, Lisa Holer, Jürg Bernhard, Tobias Hölscher, Winfried Arnold, Bülent Polat, Guido Hildebrandt, Arndt-Christian Müller, Paul Martin Putora, Alexandros Papachristofilou, Corinne Schär, Stefanie Hayoz, Marcin Sumila, Kathrin Zaugg, Matthias Guckenberger, Piet Ost, Davide Giovanni Bosetti, Christiane Reuter, Silvia Gomez, Kaouthar Khanfir, Marcus Beck, George N. Thalmann, Daniel M. Aebersold, Pirus Ghadjar

**Affiliations:** a Kantonsspital Winterthur, Winterthur, Switzerland; b Swiss Group for Clinical Cancer Research Competence Center, Bern, Switzerland; c Inselspital, Bern University Hospital, and Bern University, Bern, Switzerland; d International Breast Cancer Study Group Coordinating Center, Bern, Switzerland; e University Hospital Carl Gustav Carus, Technische Universität Dresden, Dresden, Germany; f Kantonsspital Luzern, Lucerne, Switzerland; g University Hospital Würzburg, Würzburg, Germany; h University Hospital Rostock, Rostock, Germany; i University Hospital Tübingen, Tübingen, Germany; j Kantonsspital Sankt Gallen, Sankt Gallen, Switzerland; k University Hospital Basel, Basel, Switzerland; l Hirslanden Hospital Group, Zurich, Switzerland; m Stadtspital Triemli, Zurich, Switzerland; n University Hospital Zurich, Zurich, Switzerland; o Ghent University Hospital, Ghent, Belgium; p Istituto Oncologico della Svizzera Italiana, Bellinzona, Switzerland; q Kantonsspital Münsterlingen, Münsterlingen, Switzerland; r Kantonsspital Aarau, Aarau, Switzerland; s Hôpital du Valais, Sion, Switzerland; t Charité – Universitätsmedizin Berlin, Germany

**Keywords:** Prostate Cancer, Erectile dysfunction, Salvage, Radiation Therapy, Dose intensification

## Abstract

**Objectives:**

To evaluate effects of dose intensified salvage radiotherapy (sRT) on erectile function in biochemically recurrent prostate cancer (PC) after radical prostatectomy (RP).

**Materials and methods:**

Eligible patients had evidence of biochemical failure after RP and a PSA at randomization of ≤ 2 ng/ml. Erectile dysfunction (ED) was investigated as secondary endpoint within the multicentre randomized trial (February 2011 to April 2014) in patients receiving either 64 Gy or 70 Gy sRT. ED and quality of life (QoL) were assessed using CTCAE v4.0 and the EORTC QoL questionnaires C30 and PR25 at baseline and up to 5 years after sRT.

**Results:**

344 patients were evaluable. After RP 197 (57.3 %) patients had G0-2 ED while G3 ED was recorded in 147 (42.7 %) patients. Subsequently, sexual activity and functioning was impaired. 5 years after sRT, 101 (29.4 %) patients noted G0-2 ED. During follow-up, 44.2 % of patients with baseline G3 ED showed any improvement and 61.4 % of patients with baseline G0-2 ED showed worsening. Shorter time interval between RP and start of sRT (p = 0.007) and older age at randomization (p = 0.005) were significant predictors to more baseline ED and low sexual activity in the long-term. Age (p = 0.010) and RT technique (p = 0.031) had a significant impact on occurrence of long-term ED grade 3 and worse sexual functioning. During follow-up, no differences were found in erectile function, sexual activity, and sexual functioning between the 64 Gy and 70 Gy arm.

**Conclusion:**

ED after RP is a known long-term side effect with significant impact on patients’ QoL. ED was further affected by sRT, but dose intensification of sRT showed no significant impact on erectile function recovery or prevalence of de novo ED after sRT. Age, tumor stage, prostatectomy and RT-techniques, nerve-sparing and observation time were associated with long-term erectile function outcome.

ClinicalTrials.gov. Identifier: NCT01272050.

## Highlights

• Prostate Cancer.
• Salvage Radiotherapy.
• Dose intensification.
• Erectile Dysfunction.
• Patient Reported Outcome Measures (PROMs).

## Introduction

Radical prostatectomy (RP) is one of the standard procedures for patient with localized prostate cancer (PC) and may be followed by postoperative radiotherapy (RT) to the prostate bed for patients with biochemical recurrence or adverse pathologic findings [1], [2], [3]. Three randomized controlled trials [4], [5], [6] as well as one metanalysis [7] showed a preference for early salvage (sRT) over adjuvant radiation therapy (aRT) [8], [9], [10] due to similar biochemical control but sparing half of men from pelvic RT and its associated side effects. A negative effect on erectile function has been associated with postoperative RT as compared to observation [11], [12], [13], [14]. Comparing sRT to aRT, there is evidence that delaying postoperative RT resulted in improved erectile function [6], [15], [16]. The SAKK09/10 randomized phase 3 trial was designed to assess the impact of sRT dose intensification to the prostate bed comparing 70 Gy to 64 Gy and demonstrated that conventional dose of 64 Gy was sufficient in patients with early biochemical progression of PC after RP. Dose intensified sRT increased the gastrointestinal side effects without significant differences in quality of life (QoL) [17]. In this long-term analysis, we analyzed whether dose intensified sRT impacted on erectile function [18], and report on QoL of these patients.

## Patients and methods

### Trial design and participants

The SAKK 09/10 randomized phase 3 trial on dose-intensified versus standard-dose sRT to the prostate bed in biochemically relapsed PC patients without macroscopic disease recruited patients from 28 hospitals in Switzerland, Germany, and Belgium [17]. Patients were eligible if they had evidence of biochemical failure (BF) (two consecutive rises in PSA with final PSA > 0.1 ng/ml, or 3 consecutive rises) and a PSA at randomization of ≤ 2 ng/ml.

Main inclusion and exclusion criteria have been reported [19]; for complete list see ClinicalTrials.gov (Identifier: NCT01272050). Briefly, patients were included with lymph node-negative adenocarcinoma of the prostate treated with RP at least 12 weeks before randomization with a tumor stage pT2a − 3b, R0 − 1, pN0, or cN0 who experienced biochemical progression after RP defined as two consecutive rises in PSA with the final PSA > 0.1 ng/mL or 3 consecutive rises and having a PSA at randomization ≤ 2 ng/ml. Patients with persistent PSA greater than 0.4 ng/ml 4 to 20 weeks after RP, any form of androgen deprivation therapy (ADT), macroscopic local recurrence or pelvic lymph node metastasis were excluded.

### Treatment and follow-up procedures

RP was performed at least 12 weeks before randomization and was not part of this trial. All RP techniques were permitted. sRT was administered in the standard arm to a total dose of 64 Gy in 32 fractions (2 Gy over 6.4 weeks) (arm A), and in the experimental arm to 70 Gy in 35 fractions (2 Gy over 7 weeks) (arm B). CT simulation for treatment planning was required. Patients were positioned in supine position and treated with comfortably full bladder and empty rectum. Prostate bed, clinical target volume (CTV), and planning target volume (PTV) were contoured according to the European Organisation for Research and Treatment of Cancer (EORTC) guidelines [20]. PTV was defined as CTV + 10 mm margins in all directions except for an 8–10 mm margin posteriorly. Margins were reduced for centres using image-guided sRT approved for the trial, but minimal margins around CTV were 5 mm. Dose prescription was done to the median dose D_50%_ of the PTV. Dose variation in the PTV was required to be within + 7 %/− 5 % of the prescribed dose, i.e., the 95 % − isodose encompassed the PTV.

Organs at risk (OAR) included bladder, rectum, and femoral heads. The penile bulb was not contoured as part of the study protocol requirements. The rectum was contoured from the anus to the recto-sigmoid flexure or the caudal part of the sacroiliac joint. Besides whole organ delineation, bladder wall (BW) and rectal wall (RW) were contoured using a 5 mm internal margin. Constraints for OAR were: RW: V60Gy ≤ 50 % and V70Gy ≤ 20 %; BW: V65Gy ≤ 50 %; Femoral heads: V50Gy ≤ 10 %. Megavoltage equipment with nominal photon energies ≥ 6 MV was required. Three dimensional-conformal RT (3D-CRT), intensity-modulated RT (IMRT) and rotational techniques including tomotherapy® or volumetric-modulated arc technique (VMAT) could be used. A three-step sRT QA program was carried out including a site and trial-specific questionnaire completed by the local principal investigator, a mandatory dummy run, and central archiving of all treatment plans [21].

### Erectile Dysfunction and quality of life assessment

Detailed information on trial design and primary endpoints have been described [17]. ED was assessed according to National Cancer Institute Common Terminology Criteria for Adverse Events (NCI CTCAE) v 4.0 [22]. To detect any change in erectile function, cut-off for statistical analysis was defined as patients presenting with severe ED (grade 3) (decrease in erectile function but erectile intervention not helpful, placement of a permanent penile prosthesis indicated) versus no (grade 0) or mild ED (grades 1 – 2) at baseline. For patients who presented with ED grades 1 – 3 at baseline, erectile function change was assessed three and six months after treatment, then every six months until three years after sRT and thereafter every 12 months. Correspondingly, for patients who presented with full erectile function at baseline (grade 0), a change was categorized into mild ED (grades 1––2) or severe ED (grade 3).

QoL was assessed at baseline and up to 5 years after completion of sRT, by the EORTC QLQ-C30 (version 3) [23], the PC module QLQ-PR25 [24], and an adapted indicator for overall burden [25].

### Statistical analysis

Analysis was based on the intention-to-treat (ITT) population (defined as all patients without major eligibility deviations who started sRT).

Baseline ED was compared between treatment arms using chi-squared tests. The influence of pre-selected covariates (Table S5) on baseline ED was assessed by multiple logistic regression with backward selection. ED over time was analysed by generalized mixed models with independent variables of treatment, visit and treatment-by-visit interaction. An unstructured covariance-matrix was used for the within-patient correlation modelling. Pre-selected covariates were separately added to the model.

The symptom and function scales of the QLQ-C30 and the QLQ-PR25 were scored and linearly transformed to 0–100 scales (EORTC manual). A higher score of a symptom scale or item indicates a worse condition, a higher score of a functional scale or global health status/QoL a better condition. The indicator for overall burden was linearly transformed to a 0–100 scale, with higher scores indicating greater burden. Clinically meaningful changes were defined for the QLQ-C30 according to reference data [26], [27], and for the QLQ-PR25 and overall burden according to a distribution-based measure [28]; clinically meaningful change: ≥ 3.3 in either direction; we considered the cut-off for changes of QLQ-PR25 scales as defined in the trial protocol (i.e., 10 points) as too conservative [27]. The influence of pre-selected covariates on change in sexual activity and sexual functioning over time was assessed by linear mixed models including independent variables of baseline score, treatment, visit and treatment-by-visit interaction and with unstructured covariance-matrix.

Two-tailed tests with significance level 0.05 were used for all analyses. As no adjustment for multiple testing was made, they were exploratory and hypothesis generating. All analyses were performed using SAS 9.4 (SAS Institute) and R 4.1 (https://www.r-project.org).

## Results

### Patient characteristics

Between February 2011 and April 2014, 350 patients were randomized (191 patients in Switzerland, 146 in Germany, and 13 in Belgium). Three patients (2 in the 64 Gy and 1 in the 70 Gy arm) received no sRT because of withdrawal of consent, and three (all in the 64 Gy arm) were found to be ineligible after randomization and were excluded from the ITT population, resulting in 344 patients in the ITT population [17]. Patients’ characteristics are summarized in Table 1.

**Table 1:** Patients Characteristics for the Intention-to-Treat Population (N = 344).

**Variable**	**Arm A (64 Gy)** **(N = 170)** **n (%)**	**Arm B (70 Gy)** **(N = 174)** **n (%)**
Median PSA before prostatectomy, ng/ml (IQR)	8.1 (5.4–11.6)	7.6 (5.3–12.7)
Gleason score, n (%)		
≤6	25 (15)	26 (15)
7	115 (68)	115 (66)
≥8	30 (18)	33 (19)
Tumor classification, n (%)		
pT2a	7 (4.1)	12 (6.9)
pT2b	3 (1.8)	8 (4.6)
pT2c	93 (55)	81 (47)
pT3a	49 (29)	54 (31)
pT3b	18 (11)	19 (11)
Lymphadenectomy performed (pN0), n (%)	150 (88)	151 (87)
Median number of lymph nodes removed, n (IQR)		
Left	5 (3–8)	5 (3–7)
Right	5 (3–8)	5 (3–7)
Extend of lymphadenetomy, n (%)		
Extended lymph node dissection	43 (25)	44 (25)
Limited lymph node dissection	104 (61)	105 (60)
None	20 (12)	23 (13)
Missing	3 (2)	2 (1)
Persistent PSA ≥ 0.1 ng/ml after prostatectomy, n (%)	35 (21)	35 (20)
PSA ≤ 0.5 ng/ml at randomization, n (%)	129 (76)	129 (74)
EAU high risk, n (%) ^a^	129 (76)	121 (70)
GETUG high risk, n (%) ^b^	124 (73)	121 (70)
Median age at randomization, yr (IQR)	67 (63–71)	66 (62–70)
Median time from surgery to RT start, mo (IQR)	26 (14–42)	30 (16–51)
WHO performance status 0 at treatment start, n (%)	160 (94)	161 (93)
Diagnositc imaging technique, n (%)		
Computed tomography	58 (34)	62 (36)
Magnetic resonance imaging	112 (66)	112 (64)
Prostatectomy technique, n (%)		
Laparoscopic	18 (11)	17 (10)
Perineal	4 (2)	7 (4.0)
Retropubic	116 (68)	108 (62)
Robotically assisted	32 (19)	36 (21)
Missing	0 (0)	6 (3)
Resection margins, n (%)		
R0	92 (54)	98 (56)
R1	78 (46)	76 (44)
Nerve-sparing technique, n (%)		
Bilateral	62 (36)	60 (35)
Unilateral	25 (15)	36 (20.6)
None	83 (49)	72 (41)
Missing	0 (0.0)	6 (3.4)
Radiation therapy technique, n (%)		
Three-dimensional conformal radiation therapy	74 (44)	75 (43)
IMRT	35 (21)	29 (17)
VMAT/rotational techniques	61 (35)	69 (39)

### Erectile function and quality of life at baseline

At study entry and at baseline after RP 47 (13.7 %) patients presented with full erectile function (grade 0). 150 (43.7 %) patients reported mild (grades 1 – 2) ED, 68 (45.3 %) of which were treated in arm A (64 Gy) and 82 (54.7 %) in arm B (70 Gy). 147 (42.7 %) patients suffered from severe ED (grade 3) with 78 (53.1 %) patients being treated in arm A (64 Gy) and 69 (46.9 %) patients in arm B (70 Gy). 61 patients received their sRT within 12 months after RP of which 32 (52.5 %) reported no severe ED (grade 3). In a multiple logistic regression after backward selection, time between RP and start of sRT (p = 0.007), and age at randomization (p = 0.005) were the only significant predictors, with shorter time interval and higher age leading to more baseline ED. (Table S1, Supplement).

The questionnaire submission rate was 99 % at baseline, 94 % at 3 months, 88 % at 1 year, 84 % at 2 years, 78 % at 3 and 4 years, and 70 % at 5 years. Completed questionnaires had few missing data, except for sexual functioning (Number of patients: at base line 200/344: 58.1 %, at 60 months: 84/344: 25.6 %) and overall burden (Number of patients: at base line 263/344: 76.5 %, at 60 months: 137/344: 39.8 %). Both sexual activity and functioning scores were markedly impaired at baseline, with higher activity in in Arm A (66.7) than B (50.0). Patients with higher grade of ED reported substantially worse sexual activity (37.4 vs 46.6, p = 0.001). A corresponding difference between severe ED (grade 3) versus erectile function preservation (grade 0–2) was indicated by sexual functioning (46.3 vs 58.8, p < 0.001). Patients with baseline erectile function preservation also reported slightly better physical functioning (96.3 vs 93.9, p = 0.028), but no significant differences in overall burden (32,6 vs 38,0, p = 0.09), role functioning (94.0 vs 91.1, p = 0.12) and global health status/QoL (32.6 vs 38.0, p = 0.4) were observed.

### Long-term erectile function after completing sRT

At 1, 2 and 5 years of follow-up, 164, 148 and 101 patients (47.7 %, 43.0 %, 29.4 %) reported no (grade 0) or mild ED (grades 1 and 2). An overview of the overall erectile dysfunction during follow-up can be found as Fig. S1 in the supplement.

**Fig. 1:**
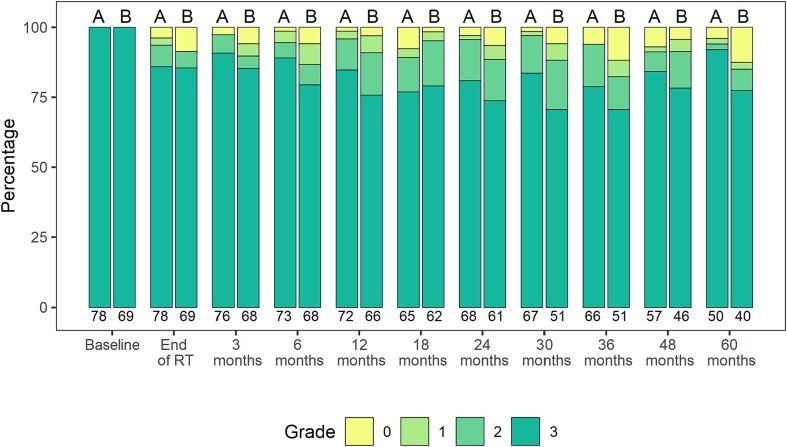
**Erectile function during follow-up in patients with baseline severe ED (grade 3)** Effect of radiation dose on erectile function during the 5 – year follow-up in patients with baseline severe ED (grade 3). Assessment of erectile function at baseline, at end of RT, at 3 months and every 6 months thereafter until 60 months. A = Study arm 64 Gy, B = Study arm 70 Gy. NCI CTCAE v4.0 grades: no (grade 0), mild erectile dysfunction (grades 1 – 2) and severe erectile dysfunction.

Of the initial 147 patients (42.7 %) with baseline severe ED (grade 3), erectile function improvement by any grade during follow-up was achieved after sRT in 65 patients (44.2 %), no significant difference was detected between treatment arms: 31 patients (39.7 %) versus 34 patients (49.3 %) (p = 0.320) (Fig. 1).

**Fig. 2:**
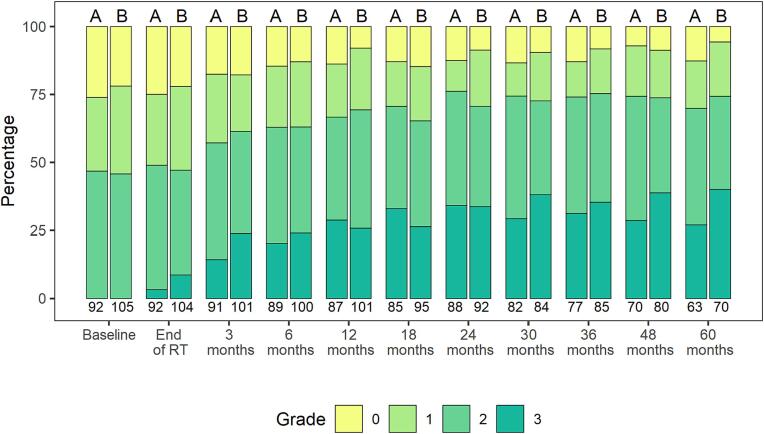
**Erectile function during follow-up in patients with baseline no or mild ED (grades 1 and 2)** Effect of radiation dose on erectile function during the 5 – year follow-up in patients with baseline no or midl ED (grades 1 and 2). Assessment of erectile function at baseline, at end of RT, at 3 months and every 6 months thereafter until 60 months. A = Study arm 64 Gy, B = Study arm 70 Gy. NCI CTCAE v4.0 grades: no (grade 0), mild erectile dysfunction (grades 1 – 2) and severe erectile dysfunction.

Of the 197 patients (57.3 %) reporting no (grade 0) or mild ED (grades 1 and 2) at baseline, the proportion of patients who showed worsening of their erectile function during follow − up was 121 patients (61.4 %) combined in both arms. In arm A (64 Gy), this corresponded to 54 patients (58.7 %) and in arm B (70 Gy) 67 patients (63.8 %), respectively. In contrast, 49 patients (24.9 %) reported an improvement in erectile function, 20 patients in Arm A (21.7 %) and 29 patients in Arm B (27.6 %). ED during the 5 – year follow-up in patients with no (grade 0) or mild ED (grades 1 and 2) at baseline is shown in Fig. 2 for both treatment arms.

Of note, after a time interval of 24 months the percentage of patients with severe ED (grade 3) did not change anymore until end of follow-up at 5 years. Similarly, ED recovery after 24 months did not improve until end of follow – up.

In the mixed model for severe ED, there was an effect over time (p < 0.001), with no significant difference between treatment arms (p = 0.321) (Table 2 and S2).

**Table 2:** Results of mixed model for severe ED (grade 3).

	**OR**	**95 % CI**		**p-value**
Intercept	0.51	0.24–1.07		0.075
Arm B	0.59	0.21–1.67		0.321
Time (Reference: Baseline)				< 0.001*
3 months	1.49	0.79–2.83		0.220
6 months	1.97	1.03–3.79		0.041
12 months	2.52	1.30–4.88		0.006
18 months	2.47	1.26–4.86		0.008
24 months	2.89	1.48–5.64		0.002
30 months	2.62	1.33–5.15		0.005
36 months	2.35	1.18–4.66		0.015
48 months	3.37	1.63–6.99		0.001
60 months	5.09	2.37–10.92		0.000

Interaction (Reference: Baseline x Arm B)				0.954*
3 months x Arm B	1.51	0.62–3.68		0.360
6 months x Arm B	0.95	0.39–2.32		0.907
12 months x Arm B	0.70	0.28–1.72		0.433
18 months x Arm B	0.91	0.36–2.30		0.846
24 months x Arm B	0.96	0.38–2.42		0.934
30 months x Arm B	1.37	0.53–3.52		0.516
36 months x Arm B	1.13	0.44–2.91		0.806
48 months x Arm B	1.22	0.45–3.29		0.701
60 months x Arm B	0.97	0.34–2.73		0.948

Number of observations	2991			
Number of patients	344			
Variance: Patients (Intercept)	14.56			

*p-value from test of effects.

### Long-term sexual activity and functioning after completing sRT

**Fig. 3:**
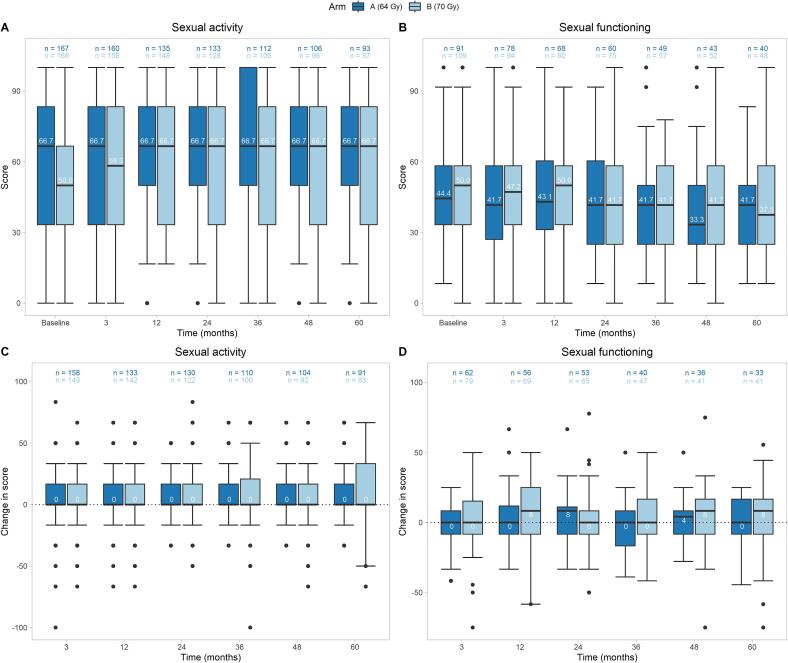
**A, B, C, D: Overall sexual activity and sexual functioning scores (A/B) and corresponding changes to baseline (C/D)** Effect of radiation dose on quality of life with respect to sexual activity and sexual functioning during the 5 – year follow-up in all patients. Assessment (Boxplot) of sexual activity score (A), change in sexual activity score (C), sexual functioning score (B) and change in sexual functioning score (D) at baseline, at 3 and 12 months and yearly thereafter until 60 months. The symptom and function scales of the QLQ-C30 and the QLQ-PR25 were scored and linearly transformed to 0–––100 scales (EORTC manual). A higher score of a symptom scale or item indicates a worse condition, a higher score of a functional scale or global health status/QoL a better condition.

Overall, the median sexual activity scores remained stable over the 5 years of follow-up, with an increase in Arm B up to 12 months (50.0 to 66.7) to the level of Arm A. Sexual functioning was likewise stable over the whole observation period. Figs. 3 A − D illustrate the scores for sexual activity and functioning and their changes to baseline with respect to ED grading 0,1,2, versus 3. Patients with ED grade 3 reported consistently *better* activity compared to the others. The magnitude of this effect was of clinical relevance over the whole observation period. It was less pronounced in functioning.

In the mixed model with change from baseline as outcome variable, for sexual activity, there was no significant difference between the treatment arms (p = 0.214) (TABLE 3). Time had an effect (p = 0.03). When adding the pre-selected covariates separately, age (p < 0.001) and time from prostatectomy to RT start (p = 0.017) had a statistically significant impact (Table S3).

**Table 3:** Results of mixed model for sexual activity and functioning.

	**Sexual activity**			**Sexual functioning**		
	**Coef**	**SE**	**p-value**	**Coef**	**SE**	**p-value**
Intercept	26.88	2.72	<0.001	21.74	3.30	<0.001
Baseline score	−0.39	0.04	<0.001	−0.46	−0.46	<0.001
Arm B (70 Gy)	−2.97	2.39	0.214	0.16	3.00	0.956

Time (Reference: 3 months)			0.030*			0.266*
12 months	1.03	1.84	0.575	1.88	2.53	0.459
24 months	2.93	1.86	0.114	−0.49	2.57	0.850
36 months	4.03	1.96	0.040	−3.31	2.82	0.240
48 months	4.41	2.00	0.028	−0.38	2.91	0.897
60 months	3.95	2.09	0.059	−1.71	3.00	0.569

Interaction (Reference: 3 months x Arm B)			0.863*			0.989*
12 months x Arm B	1.25	2.60	0.631	1.02	3.38	0.764
24 months x Arm B	1.11	2.68	0.679	0.41	3.44	0.905
36 months x Arm B	−0.28	2.86	0.922	2.54	3.80	0.504
48 months x Arm B	−2.45	2.92	0.402	1.85	3.93	0.637
60 months x Arm B	0.04	3.04	0.989	0.99	4.00	0.805

Number of observations	1414			622		
Number of patients	325			178		
Variance: Patients (Intercept)	203.86			164.69		
Variance: Residual	238.01			170.97		

*p-value from test of effects.

For sexual functioning, there was no difference by treatment (p = 0.956) and time (p = 0.266; Table 3) in the mixed model. Adding the pre-selected covariates separately, age (p = 0.021) and RT technique (p = 0.017) were statistically significant (Table S4).

## Discussion

The results from this trial comparing 64 Gy vs 70 Gy to the prostate bed showed that the use of sRT as well as sRT dose intensification affected the dynamics of erectile function from baseline to five years after sRT. Patients presenting with severe ED prior to sRT did not demonstrate signs of recovery towards milder form of ED (Fig. 1). Patients with full erectile function or mild ED prior to sRT experienced worsening of their erectile function, and number of patients with of severe ED increased over the first 12 months (Fig. 2). There was no difference in ED between men treated with sRT with 64 Gy or dose intensification to 70 Gy, and there was no change or difference in overall sexual activity or sexual functioning in both treatment arm over the 5 − year follow − up.

Maintaining sexual function is an important QoL aspect for men undergoing curative treatment for PC. In line with findings of previous studies [12], [15], [16], [29], our results showed that two-thirds of patients included in the study presenting with no or mild ED prior to sRT and treated with sRT, experienced a constant reduction in their erectile function after 1, 2 and 5 years of follow-up (Fig. 2), most likely as a consequence of sRT. After 5 years, only 25 % of patients with initial no or mild ED showed preservation or improvement of their erectile function status as prior to sRT.

A longer time interval between RP and start of sRT (>12 months vs < 12 months) as well as younger age resulted in a better erectile function prior to start of sRT. However, a longer time interval to start sRT did not impact on erectile function status after completion of sRT and during long − term follow − up. This contrasts with van Stam et al [15] and Zaffuto et al [16], demonstrating that a longer time interval (>7 months) resulted in a better erectile function after completion of sRT. In their studies observation times after sRT were up to 24–––36 months supporting erectile function recovery. Importantly, in both retrospective studies erectile function was evaluated without patient-reported outcome measurements resulting most likely in a bias in erectile function assessment [14]. Also, patient selection bias might have been relevant in their retrospective analyses [15], [16]. Timing the start of sRT after RP with respect to erectile function recovery is discussed controversially, as other groups described no or only a small improvement of erectile function with a longer time interval between RP and sRT [12], [29]. We were unable to detect such time effect after completion of sRT and long term follow up. In the GETUG-AFU 17 study comparing aRT versus sRT late erectile dysfunction grade 2 or worse was significantly higher in the aRT group than in the sRT group, favouring sRT with respect to preserve as much as erectile function as possible [6].

With regards to QoL, the time from RP to the start of sRT was statistically significant in the univariate analysis for sexual activity in the mixed model, when adding pre-selected covariates separately (Table S3). For erectile function related and general quality of life Westhofen et al found a similar statistically significant improvement with deferred RT (defined as > 6 months after surgery) as opposed to early RT [30].

Older age was weakly associated with worse baseline ED, confirming that older age favours the occurrence of ED with age also being a factor associated with ED during long-term follow up. Age has regularly been described as a prognostic factor for ED after prostate cancer therapy [31], [32], [33].

RP technique and nerve-sparing technique at time of RP had a significant impact on ED. This result is in line with findings in the retrospective study by Bastasch et al demonstrating that modern RT technique (i.e. dose escalated intensity-modulated RT to 70 Gy) had no negative effect on erectile function for patients who remained potent after bilateral nerve-sparing RP [34]. Younger men confronted with the diagnosis of localized prostate cancer and eligible for RP with nerve-sparing technique [35] should be informed by the urologist about this treatment option, as this might impact on erectile function recovery if sRT is needed. Also, radiation technique was a significant factor in this cohort. High quality data on this is somewhat scarce. General data suggests that there could be – as shown for reducing the incidence for late GU ≥ 2 toxicity [17] – a benefit of modern techniques like IMRT [36], [37], although others do not [38], [39], [40], [41], [42].

Regarding QoL, age and RT techniques along with baseline function proved to have a significant impact in this cohort. Older age as well as poor baseline function are known risk factors for impaired sexual function after treatment [36], [43], [44].

Another factor that showed a significant impact on sexual activity and sexual function was tumor classification defined as pT3b vs. other tumor stages. This might be corresponding to the extent of the previous surgery as well as the radiation field. Even if nerve sparing surgery was performed there is the possibility of bruising and trauma, that might be more pronounced in T3b disease due to the extent of surgery needed to achieve R0-resection [45].

ED represents a very common and challenging long-term side effect with profound impact on QoL in men undergoing RP and sRT [12], [14], [15], [16], [29], [46]. The data from this trial show that nearly two thirds of men with no or mild ED showed an increase in ED after sRT while a minority of patients with ED showed improved erectile function during long-term follow-up. ED recovery was very unlikely after about 2 years of completion of sRT (Fig. 1). However, patients with severe ED could maintain their sexual activity in the long-term follow – up (Fig. 3 A and C). There are several risk factors associated with worse erectile function like age, nerve sparing operating technique and RT technique. It is important that treating physicians are aware of the sexual sequelae and address these issues when discussing the impact of sRT with patients after RP and offer sexual counselling if needed [47], [48], [49].

Although this analysis was based on data collected on a randomized phase 3 trial, it is not without limitations. Information on erectile function before RP as well as during the time interval between RP and randomization of sRT is missing and this information was not mandatory for trial inclusion. Additionally, the absence of observing more differences with respect to erectile function preservation after sRT for biochemically recurrent prostate cancer after RP might be due to factors that are not included in the analysis and to factors which are ‘a priori’ unknown. A further limitation is that since this a secondary analysis of a multicentre randomized radiotherapy trial, the classification of toxicity (CTCAE) as well as measures of QoL of patients were chosen with respect to the primary endpoint of the trial. We do acknowledge that there might be other more specific and more commonly used instruments to measure ED (e.g. International index for erectile function (IIEF)) allowing additional comparative analysis with current literature [30], [39], [46]. Dose to the penile bulb was not collected and analyzed, and at the time of study design this was not seen as necessary. However, Zhang et al showed in their data from a phase 3 trial that erectile tissue sparing IMRT limiting dose to the penile bulb and corporal bodies did not show an effect on potency preservation outcomes at 2 years, highlighting the difficulty to correlate dose to penile bulb and erectile function [42]. It should be noted that the SAKK 09/10 phase 3 trial excluded patients requiring ADT at randomisation. Therefore, the impact of ADT in combination with SRT on erectile function cannot be addressed. In the context of these limitations, our findings contribute to clinical practice, given that sRT is a common treatment strategy and ED represents one of the common side effects after RP in patient with PC, whilst discussing multimodal therapies impacting on erectile function in the long term [46], [50].

## Conclusions

ED after RP is a common long term side effect with significant impact on patients’ QoL. ED was further affected by sRT, but dose intensification of sRT showed no significant impact on erectile function recovery or prevalence of de novo ED after sRT. Age, prostatectomy technique, nerve-sparing technique, tumour classification, RT technique, and time were factors associated with long term erectile function outcome. Treating physicians need to be aware of the sexual sequelae and offer sexual treatment decision counselling.

## Funding

The SAKK 09/10 trial is funded by grants provided by the Hedy and Werner Berger-Janser Foundation, Krebsforschung Schweiz (Swiss cancer research foundation), Radio-Onkologie Berner Oberland AG, Switzerland and Swiss State Secretariat for Education, Research and Innovation (SERI).

## CRediT authorship contribution statement

**Daniel R. Zwahlen:** Conceptualization, Methodology, Data curation, Writing – review & editing, Supervision, Project administration. **Christina Schröder:** Methodology, Formal analysis, Investigation, Data curation, Writing – original draft, Writing – review & editing, Visualization. **Lisa Holer:** Methodology, Formal analysis, Investigation, Data curation, Writing – original draft, Writing – review & editing, Visualization. **Jürg Bernhard:** Methodology, Formal analysis, Investigation, Data curation, Writing – original draft, Writing – review & editing, Visualization. **Tobias Hölscher:** Writing – review & editing. **Winfried Arnold:** Writing – review & editing. **Bülent Polat:** Writing – review & editing. **Guido Hildebrandt:** Writing – review & editing. **Arndt-Christian Müller:** Writing – review & editing. **Paul Martin Putora:** Writing – review & editing. **Alexandros Papachristofilou:** Writing – review & editing. **Corinne Schär:** Methodology, Formal analysis, Investigation, Data curation, Writing – original draft, Writing – review & editing, Visualization. **Stefanie Hayoz:** Methodology, Formal analysis, Investigation, Data curation, Writing – original draft, Writing – review & editing, Visualization. **Marcin Sumila:** Writing – review & editing. **Kathrin Zaugg:** Writing – review & editing. **Matthias Guckenberger:** Writing – review & editing. **Piet Ost:** Writing – review & editing. **Davide Giovanni Bosetti:** Writing – review & editing. **Christiane Reuter:** Writing – review & editing. **Silvia Gomez:** Writing – review & editing. **Kaouthar Khanfir:** Writing – review & editing. **Marcus Beck:** Writing – review & editing. **George N. Thalmann:** Writing – review & editing. **Daniel M. Aebersold:** Writing – review & editing. **Pirus Ghadjar:** Writing – review & editing.

## Declaration of Competing Interest

The authors declare the following financial interests/personal relationships which may be considered as potential competing interests: ED after RP is a common long term side effect with significant impact on patients’ QoL. ED was further affected by sRT, but dose intensification of sRT showed no significant impact on erectile function recovery or prevalence of de novo ED after sRT. Age, prostatectomy technique, nerve-sparing technique, tumour classification, RT technique, and time were factors associated with long term erectile function outcome. Treating physicians need to be aware of the sexual sequelae and offer sexual treatment decision counselling.
