# Supplementary material for: Erectile function preservation after salvage radiation therapy for biochemically recurrent prostate cancer after prostatectomy: Five-year results of the SAKK 09/10 randomized phase 3 trial
Source: Clin Transl Radiat Oncol. 2024 Apr 25;47:100786. doi: 10.1016/j.ctro.2024.100786 (PMC11067361; doi:10.1016/j.ctro.2024.100786)
Supplement: Supplementary Data 1 [file mmc1.docx]

**Supplement**

**FIGURE S1: Erectile function during follow-up in all patients**


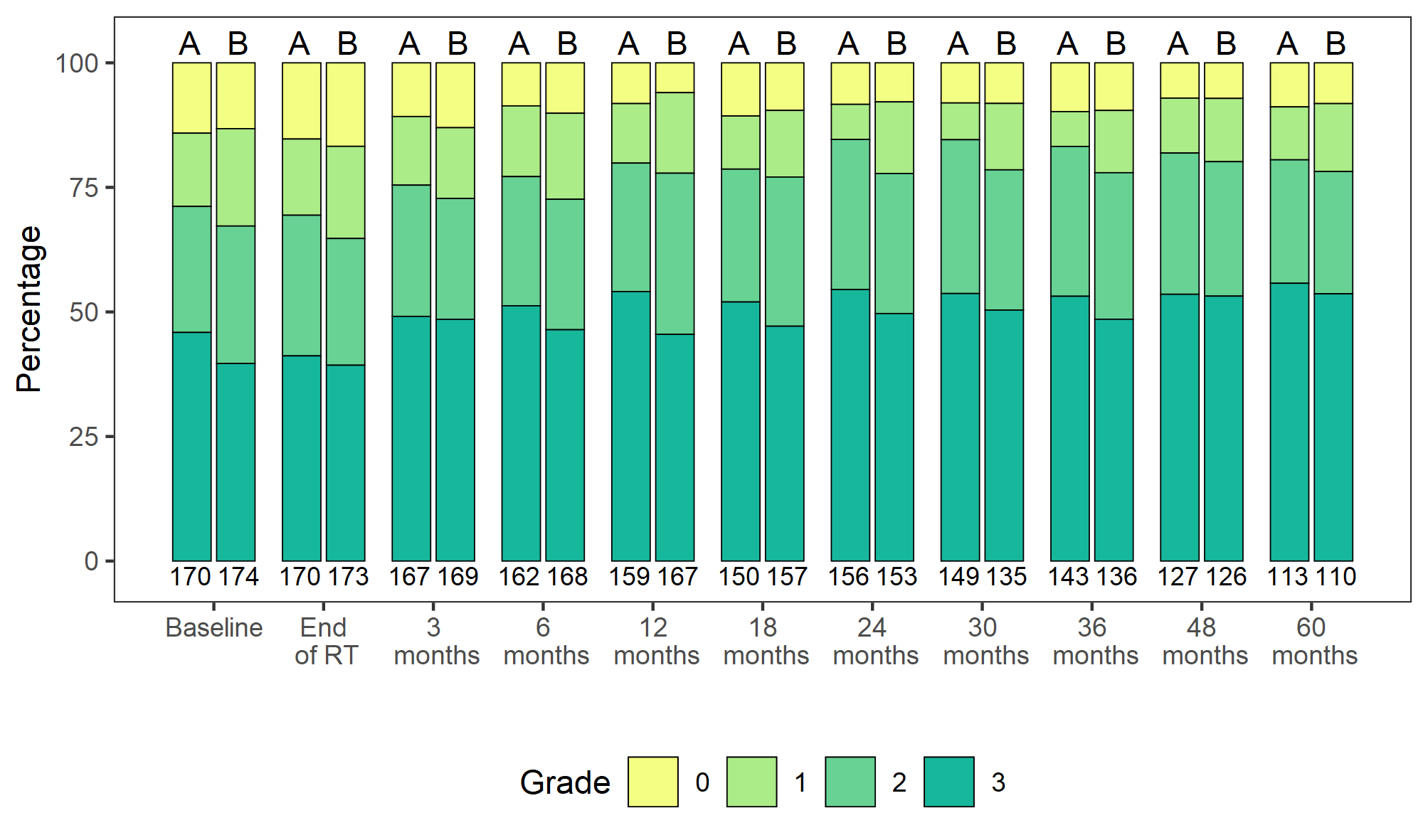


Effect of radiation dose on erectile function during the 5 – year follow-up in all patients. Assessment of erectile function at baseline, at end of RT, at 3 months and every 6 months thereafter until 60 months. A = Study arm 64 Gy, B = Study arm 70 Gy. NCI CTCAE v4.0 grades: no (grade 0), mild erectile dysfunction (grades 1 – 2) and severe erectile dysfunction.

**TABLE S1: Results of multiple logistic regression (after backward selection with significance level 0.05)**

|  | OR | 95% CI | p-value* |
| --- | --- | --- | --- |
| Intercept |  |  | 0.006 |
| Time from prostatectomy to treatment start** (days) | 0.71 | 0.55-0.91 | 0.007 |
| Age at randomization (years) | 1.06 | 1.02-1.11 | 0.005 |

*Abbreviations: ED=Erectile dysfunction; *Wald Chi-squared test; **IQR-normalized for easier interpretation*

**TABLE S2: Results (only p-values) of mixed models for severe ED (grade 3); every line represents the results of one model**

|  | **Variable** | **Time** | **Arm** | **Time * Arm** |
| --- | --- | --- | --- | --- |
| Age | 0.010 | < 0.001 | 0.334 | 0.954 |
| Weight | 0.332 | < 0.001 | 0.268 | 0.955 |
| BMI | 0.512 | < 0.001 | 0.273 | 0.934 |
| Prostatectomy technique | 0.024 | < 0.001 | 0.341 | 0.958 |
| Nerve-sparing technique | < 0.001 | < 0.001 | 0.272 | 0.970 |
| Gleason Score:  7, ≤ 6, ≥ 8 | 0.070 | < 0.001 | 0.276 | 0.953 |
| Tumor classification: other, pT3b | 0.709 | < 0.001 | 0.291 | 0.954 |
| Lymphadenectomy performed: No (cN0), Yes (pN0) | 0.114 | < 0.001 | 0.316 | 0.953 |
| Extend of lymphadenectomy | 0.183 | < 0.001 | 0.311 | 0.952 |
| Time from prostatectomy to treatment start | 0.317 | < 0.001 | 0.361 | 0.954 |
| RT technique | 0.031 | < 0.001 | 0.334 | 0.955 |
| WHO performance status at treatment start | 0.665 | < 0.001 | 0.336 | 0.966 |
| Median dose to PTV (Gy) | 0.150 | < 0.001 | 0.525 | 0.936 |
| Mean dose to PTV (Gy) | 0.254 | < 0.001 | 0.403 | 0.958 |
| Minimal dose to PTV (Gy) | 0.536 | < 0.001 | 0.257 | 0.951 |
| Maximal dose to PTV (Gy) | 0.730 | < 0.001 | 0.434 | 0.950 |
| Relative volume % to rectal wall (V60Gy) | 0.576 | < 0.001 | 0.253 | 0.954 |
| Relative volume % to rectal wall (V70Gy) | 0.366 | < 0.001 | 0.213 | 0.969 |
| Relative volume % to bladder wall (V65Gy) | 0.457 | < 0.001 | 0.201 | 0.954 |

**TABLE S3:** **Results (only p-values) of mixed models for sexual activity; every row represents the results of one model**

| **Variable** | **Baseline score** | **Variable** | **Time** | **Arm** | **Time * Arm** |
| --- | --- | --- | --- | --- | --- |
| Age | < 0.001 | < 0.001 | 0.025 | 0.137 | 0.869 |
| Weight | < 0.001 | 0.416 | 0.023 | 0.140 | 0.881 |
| BMI | < 0.001 | 0.537 | 0.020 | 0.129 | 0.864 |
| Prostatectomy technique | < 0.001 | 0.575 | 0.037 | 0.085 | 0.829 |
| Nerve-sparing technique | < 0.001 | 0.626 | 0.024 | 0.100 | 0.859 |
| Gleason Score:  7,  ≤ 6, ≥ 8 | < 0.001 | 0.341 | 0.028 | 0.100 | 0.864 |
| Tumor classification: other, pT3b | < 0.001 | 0.959 | 0.030 | 0.104 | 0.863 |
| Lymphadenectomy performed: No (cN0), Yes (pN0) | < 0.001 | 0.874 | 0.030 | 0.104 | 0.863 |
| Extend of lymphadenectomy | < 0.001 | 0.809 | 0.033 | 0.170 | 0.912 |
| Time from prostatectomy to treatment start | < 0.001 | 0.017 | 0.029 | 0.073 | 0.860 |
| RT technique | < 0.001 | 0.371 | 0.017 | 0.101 | 0.874 |
| WHO performance status at treatment start | < 0.001 | 0.870 | 0.034 | 0.097 | 0.864 |
| Median dose to PTV (Gy) | < 0.001 | 0.977 | 0.029 | 0.190 | 0.968 |
| Mean dose to PTV (Gy) | < 0.001 | 0.167 | 0.030 | 0.422 | 0.807 |
| Minimal dose to PTV (Gy) | < 0.001 | 0.317 | 0.033 | 0.469 | 0.807 |
| Maximal dose to PTV (Gy) | < 0.001 | 0.866 | 0.031 | 0.410 | 0.809 |
| Relative volume % to rectal wall (V60Gy) | < 0.001 | 0.393 | 0.032 | 0.155 | 0.860 |
| Relative volume % to rectal wall (V70Gy) | < 0.001 | 0.495 | 0.049 | 0.153 | 0.898 |
| Relative volume % to bladder wall (V65Gy) | < 0.001 | 0.896 | 0.030 | 0.200 | 0.863 |

**TABLE S4: Results (only p-values) of mixed models for sexual functioning; every row represents the results of one model**

| **Variable** | **Baseline score** | **Variable** | **Time** | **Arm** | **Time *Arm** |
| --- | --- | --- | --- | --- | --- |
| Age | < 0.001 | 0.021 | 0.252 | 0.635 | 0.991 |
| Weight | < 0.001 | 0.165 | 0.281 | 0.553 | 0.986 |
| BMI | < 0.001 | 0.100 | 0.232 | 0.658 | 0.984 |
| Prostatectomy technique | < 0.001 | 0.049 | 0.228 | 0.328 | 0.978 |
| Nerve-sparing technique | < 0.001 | 0.374 | 0.285 | 0.529 | 0.983 |
| Gleason Score:  7, ≤ 6, ≥ 8 | < 0.001 | 0.410 | 0.282 | 0.559 | 0.990 |
| Tumor classification: other, pT3b | < 0.001 | 0.134 | 0.289 | 0.522 | 0.991 |
| Lymphadenectomy performed: No (cN0), Yes (pN0) | < 0.001 | 0.069 | 0.253 | 0.559 | 0.987 |
| Extend of lymphadenectomy | < 0.001 | 0.173 | 0.243 | 0.559 | 0.990 |
| Time from prostatectomy to treatment start | < 0.001 | 0.289 | 0.268 | 0.519 | 0.989 |
| RT technique | < 0.001 | 0.017 | 0.268 | 0.542 | 0.989 |
| WHO performance status at treatment start | < 0.001 | 0.464 | 0.269 | 0.551 | 0.990 |
| Median dose to PTV (Gy) | < 0.001 | 0.418 | 0.350 | 0.447 | 0.934 |
| Mean dose to PTV (Gy) | < 0.001 | 0.465 | 0.243 | 0.412 | 0.986 |
| Minimal dose to PTV (Gy) | < 0.001 | 0.503 | 0.280 | 0.398 | 0.986 |
| Maximal dose to PTV (Gy) | < 0.001 | 0.874 | 0.279 | 0.690 | 0.986 |
| Relative volume % to rectal wall (V60Gy) | < 0.001 | 0.136 | 0.257 | 0.937 | 0.989 |
| Relative volume % to rectal wall (V70Gy) | < 0.001 | 0.630 | 0.267 | 0.517 | 0.988 |
| Relative volume % to bladder wall (V65Gy) | < 0.001 | 0.585 | 0.262 | 0.435 | 0.990 |

**TABLE S5: Predefined covariables used for univariate models**

| **Variable** |
| --- |
| Age |
| Weight |
| BMI |
| Prostatectomy technique |
| Nerve-sparing technique |
| Gleason Score:  7, ≤ 6, ≥ 8 |
| Tumor classification: other, pT3b |
| Lymphadenectomy performed: No (cN0), Yes (pN0) |
| Extend of lymphadenectomy |
| Time from prostatectomy to treatment start |
| RT technique |
| WHO performance status at treatment start |
| Median dose to PTV (Gy) |
| Mean dose to PTV (Gy) |
| Minimal dose to PTV (Gy) |
| Maximal dose to PTV (Gy) |
| Relative volume % to rectal wall (V60Gy) |
| Relative volume % to rectal wall (V70Gy) |
| Relative volume % to bladder wall (V65Gy) |

Remark: In all models the baseline score of the respective QoL score was included as covariate. For multivariate models, weight, median dose to PTV, minimal dose to PTV, maximal dose to PTV, relative volume % to rectal wall (V70Gy) and relative volume % to bladder wall (V65Gy) were removed due to collinearity.
